# Typical and Atypical Inducers of Lysosomal Cell Death: A Promising Anticancer Strategy

**DOI:** 10.3390/ijms19082256

**Published:** 2018-08-01

**Authors:** Antoni Domagala, Klaudyna Fidyt, Malgorzata Bobrowicz, Joanna Stachura, Kacper Szczygiel, Malgorzata Firczuk

**Affiliations:** 1 Department of Immunology, Medical University of Warsaw, 02-097 Warsaw, Poland; antoni.domagala@wum.edu.pl (A.D.); klaudyna.fidyt@wum.edu.pl (K.F.); malgorzata.bobrowicz@wum.edu.pl (M.B.); joanna.stachura@wum.edu.pl (J.S.); kacper.jan.szczygiel@gmail.com (K.S.); 2 Department of Urology, Holycross Cancer Center, 25-734 Kielce, Poland; 3 Postgraduate School of Molecular Medicine, 02-091 Warsaw, Poland

**Keywords:** lysosomes, lysosomal membrane permeabilization, lysosomotropic agents, autophagy, apoptosis, drug resistance

## Abstract

Lysosomes are conservative organelles with an indispensable role in cellular degradation and the recycling of macromolecules. However, in light of recent findings, it has emerged that the role of lysosomes in cancer cells extends far beyond cellular catabolism and includes a variety of cellular pathways, such as proliferation, metastatic potential, and drug resistance. It has been well described that malignant transformation leads to alterations in lysosomal structure and function, which, paradoxically, renders cancer cells more sensitive to lysosomal destabilization. Furthermore, lysosomes are implicated in the regulation and execution of cell death in response to diverse stimuli and it has been shown that lysosome-dependent cell death can be utilized to overcome apoptosis and drug resistance. Thus, the purpose of this review is to characterize the role of lysosome in cancer therapy and to describe how these organelles impact treatment resistance. We summarized the characteristics of typical inducers of lysosomal cell death, which exert its function primarily via alterations in the lysosomal compartment. The review also presents other anticancer agents with the predominant mechanism of action different from lysosomal destabilization, the activity of which is influenced by lysosomal signaling, including classical chemotherapeutics, kinase inhibitors, monoclonal antibodies, as well as photodynamic therapy.

## 1. Introduction

Lysosomes are membrane-enclosed vesicles with an indispensable catabolic role. However, in light of recent findings, it is well known that the role of lysosomes is far more complex and multifaceted. Apparently, lysosomes are not only cells’ waste bag, but important regulators of a number of cellular processes, including cell growth, adhesion, migration, autophagy, apoptosis, and other modes of cell death.

Malignant transformation leads to changes in lysosomal size, content, subcellular localization, and function. Alterations in lysosomal compartment render cancer cells more sensitive to lysosome-targeting agents [1,2,3], which offer possibility for specific tumor eradication. What is more, some reports also suggest that lysosome-targeting agents may overcome therapy resistance. In this review, we would like to summarize anticancer therapeutic strategies with the mechanism of action dependent on lysosomal compartment.

### 1.1. The Structure, Function, and Biogenesis of Lysosomes

Lysosomes, initially described as cellular “suicide bags”, are membrane-enclosed organelles responsible for the degradation of various biomolecules, such as proteins, lipids, carbohydrates, and nucleic acids. These intracellular vesicles are present in almost all eukaryotic cells and contain over 60 hydrolases, including lysosomal proteases cathepsins. To protect other cellular compartments from enzymatic digestion, the hydrolases are active mainly in acidic environment (pH ~ 4.5), which is maintained inside lysosomes by vacuolar-type H^+^ ATPases (V-ATPases) [4]. Additionally, lysosomal enzymes are detained inside the vesicles by lipid bilayer stabilized by lysosomal membrane proteins, such as lysosome-associated membrane protein 1 and 2 (LAMP1, LAMP2), lysosomal integral membrane protein 2 (LIMP2), CD63, as well as molecular chaperone heat shock protein 70 (HSP70) [5,6].

Lysosomes function as cellular digestive organelles, providing nutrient supply. Biomolecules from the outside of the cell reach the lysosome via endocytosis and phagocytosis while endogenous cargos are delivered through all types of autophagy [7]. During autophagy, damaged or obsolete organelles and macromolecules are sequestered into double-membraned vesicles termed autophagosomes, which then fuse with lysosomes to form autolysosomes. Subsequently, lysosomal hydrolases degrade autophagy cargo, which enables recycling of nutrients [8].

Coordinated Lysosomal Expression and Regulation machinery (CLEAR) tightly controls lysosomal biogenesis and function at the transcriptional level and transcription factor EB (TFEB) represents a major component of this network [9]. It is worth mentioning that lysosomes play a central role in nutrient sensing through interaction with the mechanistic target of rapamycin complex 1 (mTORC1), which is known to be a master regulator of cellular growth and proliferation [10]. This notion is further supported by the observations that mTORC1 exerts its function directly from the lysosomal membrane [11]. Moreover, it has been recently postulated that lysosomal membrane damage promotes autophagic response through mTOR inhibition [12].

### 1.2. Lysosomal Alterations in Cancer

Due to increased metabolic demands, cancer cells upregulate their lysosomal function [13]. Furthermore, lysosomal proteases—cathepsins—are involved in tumor invasion and progression [14]. As a result of high lysosomal reliance, alterations in lysosome structure render malignant cells more sensitive to the destabilization of these organelles [15]. These alterations include changes in protein and sphingolipid composition of lysosomal membranes. As an example, oncogenic transformation drives cathepsin-dependent degradation of LAMP1 and LAMP2, thus increasing the fragility of lysosomal compartment [16]. Additionally, increased lysosomal fragility observed in tumor cells is also dependent on decreased activity of acid sphingomyelinase (ASM) and subsequent rise in lysosome-destabilizing sphingomyelin [1]. Another example of altered sphingolipid content in lysosomes has been reported in chronic lymphocytic leukemia (CLL). Compared to healthy B-lymphocytes, elevated levels of sphingosine render CLL cells more prone to lysosome perturbation [17]. Sphingosine can also be converted by two sphingosine-kinase isoforms (SPHK1 and SPHK2) to form sphingosine-1-phosphate, which generally exerts antiapoptotic and prosurvival properties [18]. Overall, changes in lysosome structure in cancer cells sensitize them to LMP and may result in cell death. However, knowledge on the lysosome structure in specific types of cancers is limited and further studies are needed to identify cancer-specific alterations in lysosomes.

## 2. The Definition and Mechanisms of Lysosome-Dependent Cell Death

**Figure 1:**
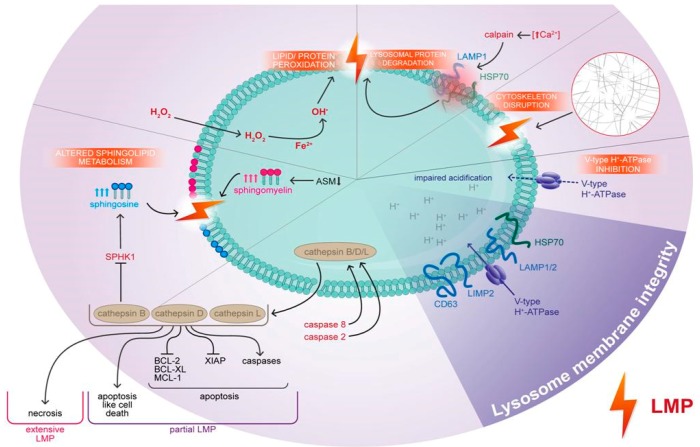
Triggers of lysosomal membrane permeabilization. Lysosome membrane integrity is protected by heat shock protein 70 (HSP70) as well as lysosomal-associated membrane protein 1/2 (LAMP1/2), lysosome integral membrane protein 2 (LIMP2) and CD63. Accordingly, degradation of LAMP1 and HSP70 leads to lysosome membrane permeabilization (LMP). LMP may be also induced by other stimuli, including ROS (H2O2), proteases such as caspases and calpains, cytoskeleton disruption and changes in sphingolipids composition in lysososmal membrane, e.g., increase in sphingomyelin and sphingosine. Inhibition of V-type H^+^ ATPase and therefore impaired acidification of the lysosome is also contributing to its destabilization. As a result of these events, the breakdown of lysosomal membrane provokes cathepsins release and subsequently lead to cell death. Detailed characteristic of LMP promoting mechanisms are described in paragraph 2. The LMP-inducing mechanisms are displayed in rectangles.

Accumulating evidence indicates that the lysosomal compartment is involved in shaping cell death in response to various internal and external stimuli, acting either as an initiator or amplifier of cell death signaling [19]. According to the definition provided by the Committee on Cell Death [20], lysosome-dependent cell death represents a form of regulated cell death initiated primarily by lysosomal membrane permeabilization (LMP). LMP involves the relocation of lysosomal constituents into the cytosol [21], which in turn triggers a whole cascade of events leading to cell death (Figure 1). The precise molecular mechanism of LMP is still unclear. It remains elusive whether LMP involves unselective destabilization of lysosomal integrity or formation of specific pores to allow selective passage of constituents.

Loss of lysosomal membrane integrity and subsequent LMP results from disruption of lysosomal lipids and proteins. It is well known that the lysosomal membrane is prone to oxidative damage [22]. Reactive oxygen species (ROS) have been shown to stimulate LMP via macromolecule peroxidation. Lysosomes are rich in redox-active iron (Fe^2+^), catalyzing a nonenzymatic Fenton reaction with hydrogen peroxide, which diffuses across the lysosomal membrane [23]. As a result, highly reactive hydroxyl radicals lead to lipid and protein peroxidation and subsequent LMP [24]. The contribution of ROS to lysosomal injury is further corroborated by the fact that LMP can be reversed by lipid-soluble scavengers, including α-tocopherol [25], which inhibit lipid peroxidation. However, as α-tocopherol incorporates into the lysosomal bilayer, inhibition of LMP may result from physical stabilization of lysosomal membrane rather than from its antioxidant properties [26].

LMP can also be triggered by cleavage and disruption of the lysosomal membrane proteins by cytosolic proteases. Calcium-activated proteases, calpains, were reported to promote LMP via cleavage of HSP70 [27] and LAMP1 [28], whereas caspases, in particular, caspase 8 and caspase 2 have been noted to induce cathepsins translocation into the cytosol [29,30].

Lysosomal integrity is also affected by sphingolipid composition and hence is regulated by sphingolipid-metabolizing enzymes. Specifically, sphingomyelin accumulation and inactivating mutations in sphingomyelin hydrolase—ASM—are associated with lysosomal destabilization and lysosomal storage diseases [31]. ASM resides inside lysosomal lumen and its hydrolytic activity is stabilized by a docking lipid, bis(monoacylglycero)phosphate (BMP) [32]. The chaperone protein, HSP70, further supports the interaction between ASM and BMP and thus confers resistance against lysosomal destabilization [33]. Moreover, increased activity of acid ceramidase, and subsequent upregulation of sphingosine production, may also trigger LMP.

Enlargement of lysosomes and their destabilization may also result from disruption of the cytoskeleton, which guides lysosomal turnover by exocytosis and autophagy. Vincristine, a microtubule-targeting drug, increased lysosomal compartment and triggered LMP [25]. Similarly, relocalization of actin filaments caused expansion of lysosomes and subsequent LMP [34].

The consequences of LMP and subsequent mode of cell death depend on the extent of the lysosomal damage. Complete lysosomal rupture and massive release of lysosomal hydrolases lead to uncontrolled damage of cytoplasmic components and necrosis [35]. Conversely, partial or gradual LMP provokes apoptosis, both in caspase-dependent [36] and independent manner [37].

Although lysosomal cathepsins preferentially work in acidic conditions, they may retain some activity in neutral pH [38]. Upon LMP and translocation to the cytosol, cathepsins stimulate apoptosis directly through mitochondrial depolarization or indirectly via truncation of Bid, which triggers the release of mitochondrial cytochrome c [36] and other apoptogenic factors [39]. On the other hand, intrinsic apoptosis may also trigger LMP. It was shown that mitochondrial depolarization and increased generation of ROS trigger LMP through a mechanism that involves lipid peroxidation [40]. Moreover, more recent reports imply that the lysosomal compartment is also associated with alternative ways of cellular demise, including ferroptosis [41]. The triggers of LMP and the crosstalk between various cell death pathways associated with LMP are presented schematically in Figure 1.

## 3. Lysosome-Targeting Agents as Anticancer Drugs

Several groups of agents can induce LMP and lead to lysosomal cell death (LCD) via different mechanisms. These agents can be divided into two groups: typical and atypical inducers of LCD. As for the typical inducers of LCD, the predominant mechanism of action focuses on the lysosomal compartment. Atypical inducers of LCD involve various anticancer therapeutics with the major mechanisms of action different from LCD, for which lysosomes were shown to contribute to their overall cytotoxicity. We will discuss each of these two groups of agents separately (Table 1 and Table 2).

### 3.1. Typical Lysosome-Targeting Agents

Among the typical inducers of LMP, four groups with different mechanisms of activity can be distinguished: chloroquine (CQ) and its derivatives, V-type H^+^ ATPase inhibitors, agents interfering with sphingolipid metabolism, and antagonists of HSP70.

#### 3.1.1. Chloroquine (CQ) and Its Derivatives

CQ and its derivative, hydroxychloroquine (HCQ), are widely applied in the clinical setting to treat malaria and some of the rheumatoid disorders. Moreover, CQ and HCQ are well known as autophagy inhibitors [42]. Though the exact molecular mechanism of action of CQ remains elusive, it is suggested that CQ prevents endosomal acidification [43] and therefore blocks autophagic flux by preventing cleavage of lysosomal cargo [42]. Additionally, it has been reported that HCQ, apart from raising lysosomal pH, elicits LMP [41]. The importance of the latter mechanism has been demonstrated in a study, in which CQ significantly delayed the development of Burkitt’s lymphoma via induction of LMP-dependent cell death [44]. These results are in line with the observations from the clinical trial performed in equatorial Africa, where the use of CQ decreased by 75% the incidence rate of Burkitt’s lymphoma, which reached its baseline two years after the end of the study [45].

Promising results of preclinical studies prompted the initiation of numerous clinical trials aiming to assess the utility of HCQ in cancer therapy, which has been summarized in detail elsewhere [46]. However, despite the ability of HCQ to inhibit autophagy in patients, as evidenced by autophagosome accumulation on peripheral blood mononuclear and tumor cells [47], its application in the clinical setting is limited by poor pharmacokinetics and frequent side effects [48]. Therefore, huge effort has been made to design more potent autophagy inhibitors with reduced side effects, such as Lys05, dimeric CQ derivate, which has been shown to elicit more potent autophagy inhibition [49]. Despite improved pharmacokinetic profile in comparison to HCQ, Lys05 has not been tested in clinical trials yet.

#### 3.1.2. V-Type H^+^ ATPase Inhibitors

V-type H^+^ ATPase, an ATP-dependent proton pump, maintains a low pH, which is essential for proper lysosomal functioning [71]. The disruption of lysosomal acidification was linked to LCD. A classic V-type H^+^ ATPase inhibitor, bafilomycin A1, was noted to induce cell death in tumor cell lines via the mechanism involving cathepsin leakage [53]. Lysosome-destabilizing properties were also reported for proton pump inhibitors (PPI), like omeprazole, which are commonly used clinically to treat gastric-related disorders. It has been reported that PPI lead to increased ROS generation, lysosomal destabilization, and subsequent cell death, which could be prevented by ROS scavengers. Nevertheless, the exact molecular mechanism of PPI-mediated ROS generation remains elusive [37].

#### 3.1.3. Sphingolipid Metabolism Targeting Drugs

LMP can be achieved by targeting sphingolipid metabolism at different levels; nonetheless, most of the drugs are inhibitors of ASM [72]. ASM can be targeted by many clinically applicable drugs, including antidepressants, antiarrhythmics, and antihistamines, which are collectively termed cationic amphiphilic drugs (CADs) [73]. CADs diffuse across the lysosomal membrane and become protonated inside lysosomal acidic environment. The most prominent feature of CADs is their ability to displace ASM from its docking molecule, BMP, thus leading to lysosomal degradation of ASM and buildup of sphingomyelin in lysosomes [1,33].

Among CADs, siramesine has been the most frequently studied and utilized in various in vitro and in vivo studies. Although most of the papers focus on LMP-promoting properties of siramesine [25,74], one report implies that higher concentrations of this drug can also destabilize mitochondria [75]. Application of siramesine seems attractive from a therapeutic perspective since it preferentially targets cancer cells [1]. A recent study revealed that siramesine selectively kills leukemic cells as compared to healthy B-cells [17]. Siramesine has also been tested in combination with other drugs, showing synergism in combination with vincristine [25] and lapatinib [41]. Likewise, LCD has been also observed with other CADs, including antidepressant desipramine, which showed efficacy in CLL [17]. Altogether, it should be noted that CADs hold great potential since most of them are already applied in the clinical setting and have a well-characterized safety and pharmacokinetic profile. However, none of them has been tested in cancer patients thus far.

LMP can be also achieved by increasing sphingosine composition within the lysosomal bilayer [76]. Inhibition of sphingosine kinase increases the ratio sphingosine/sphingosine-phosphate and thus augments the tendency toward LMP [77,78]. Accordingly, cathepsin B-mediated cleavage of sphingosine-kinase 1 [79] and inhibition of sphingosine-kinase 2 by the selective inhibitor, opaganib (ABC294640), induced cell death associated with alterations in lysosomal compartment [80]. Opaganib, which is orally available, shows potent antitumor activity in various cancer models [58,81,82,83] and is now tested in stage II clinical trials in patients with multiple myeloma and liver cancer [84].

#### 3.1.4. Antagonists of HSP70

HSP70 is a molecular chaperone that prevents LMP and stabilizes lysosomal membrane proteins in response to various stressful stimuli [85]. Furthermore, HSP70 stabilizes lysosomes via upregulation of ASM activity [1,33]. Both genetic [86] and pharmacological [55] inhibition of HSP70 were described to induce LMP. Pifithrin-µ (2-phenylethynesulfonamide), an HSP70 inhibitor, induced LMP in primary effusion lymphoma cell lines in vitro, but also activated dendritic cells, proving immunogenic potential [55]. Moreover, pifithrin-µ was reported to be a potent autophagy inhibitor [87], which could be potentially utilized to enhance the efficacy of other therapeutic regimens, especially those associated with cytoprotective autophagy induction.

### 3.2. Atypical LMP Inducers Utilized in Cancer Treatment

A variety of anticancer schemes can trigger LMP, albeit the significance of this phenomenon depends on the context and cell type. Thus, the following section describes the anticancer regimens with various mechanisms of actions, which are accompanied by LMP or other alterations in lysosomal functioning. Below we describe these therapeutics, referred to as atypical inducers of LCD, with the major focus on the clinically applicable therapeutic regimens.

#### 3.2.1. Classical Chemotherapeutics

A big class of long-known chemotherapeutics, which destroys cancer cells by interference with the cytoskeleton, also affects the stability of the lysosomal membrane [25,88,89]. Vincristine-mediated microtubule destabilization blocks lysosomal trafficking and consequently results in LCD, which could be further potentiated by adding siramesine [25]. Likewise, LCD was also observed with taxanes [90], a group of chemotherapeutics known to inhibit microtubule breakdown [89]. Indeed, LMP was noted to mediate docetaxel cytotoxicity in hormone-refractory prostate cancer [59].

Moreover, a lysosomotropic mechanism of action has been recently attributed to fludarabine, a commonly used nucleoside analogue. Incubation of CLL primary cells with fludarabine led to the lysosome integrity loss and cathepsin B release, which was further potentiated by the addition of valproic acid, a well-known histone deacetylase (HDAC) inhibitor that increased cathepsin B expression [60]. Similarly, cisplatin was also reported to induce cell death associated with LMP [61], whereas cisplatin resistance could be overcome by inducing LMP with CQ coincubation [91]. In addition, LMP was also attributed to the proteasome inhibitor, bortezomib, with a mechanism involving cathepsin-mediated caspase 2 activation [62].

#### 3.2.2. Thyrosine Kinase Inhibitors (TKIs) and BH3-Mimetics

TKIs are known to affect lysosome stability via several mechanisms. It has been reported that BCR-ABL inhibitor, imatinib, leads to LMP and cathepsin B redistribution into the cytoplasm, which contributs to CML eradication [63]. Moreover, lysosomes are postulated to mediate cell death after sunitinib treatment, which results from ASM inhibition [92].

BH3-mimetics represent small-molecule inhibitors of antiapoptotic proteins of the BCL-2 superfamily, which are best known to elicit classic apoptotic response [93]. However, there are reports suggesting that BH3-mimetics can influence the structure and the function of lysosomes. BCL-2 inhibitor, obatoclax, leads to lysosomal alkalization and subsequently impairs cathepsin activity [65,92,93,94,95]. Obatoclax-dependent lysosome destabilization blocks autophagosome degradation; thus, combination with other lysosome-destabilizing agents can further potentiate its efficacy [65].

#### 3.2.3. Monoclonal Antibodies (mAbs)

The lysosome-dependent mechanism of action has been also reported for some monoclonal antibodies (mAbs), which target tumor antigens on malignant cells. mAbs exert their function by triggering an immune response, which is dependent on their fragment crystallizable (Fc) regions mechanisms, i.e., induction of antibody-dependent or complement-dependent cellular cytotoxicity, as well as immunophagocytosis [96]. However, some of the mAbs, e.g., anti-CD20 mAb-obinutuzumab, induce direct LCD via a unique mechanism characterized by the homotypic aggregation of the cells. This phenomenon starts upon mAb-mediated cross-linking of cells, which induces peripheral translocation of actin filaments and rapidly drives lysosomes to this region. Consequently, lysosome membranes are damaged and the released content leads to the activation of nicotinamide adenine dinucleotide phosphate (NADPH) oxidase resulting in massive ROS production and cell death [34]. The mode of cell death evoked by obinutuzumab is nonapoptotic, Bcl-2 independent, with no signs of poly (ADP-ribose) polymerase (PARP) nor caspase cleavage [66]. The mechanisms of homotypic aggregation-initiated cell death have been best studied for obinutuzumab, albeit similar mechanism is described for some other mAbs as listed in Table 2. Lysosomal-dependent nonapoptotic cell death provides a new chance of overcoming resistance mechanisms in hematological malignancies. To our knowledge, mAbs were not combined with other LCD inducers thus far, but it would be interesting to test whether these combinations could elicit synergistic responses.

#### 3.2.4. Photodynamic Therapy (PDT)

Another strategy to elicit LMP involves PDT. Mechanistically, the mechanism of action of PDT depends on ROS generation, following photosensitizer activation by light [97]. It has been reported that photodamage of lysosomal membranes and subsequent leakage of hydrolases results in cell death, however, the mode of cell death depends on the degree of the cellular injury [98]. It is worth noting that PDT-mediated LMP occurs with the photosensitizers that preferentially accumulate in lysosomes [99,100]. Nevertheless, cancer cells differ in their sensitivity to the lysosomal photodamage, which can depend on the content and the activity of lysosomal hydrolases [101]. Moreover, it has been suggested that LMP can also prevent lysosome-dependent autophagosome degradation, and consequently blocks autophagic flux [102]. In the context of PDT, autophagy plays a dual role, having both tumor-promoting and tumor-suppressing properties, which is context-dependent [103]. Literature data suggest that PDT-induced autophagosome accumulation can eventually contribute to cell death [99], which is referred to as autophagic cell death [102]. On the other hand, autophagy was also noted to exert cytoprotective function, for example by aiding in the clearance of oxidatively-damaged proteins [104].

## 4. Lysosome-Mediated Drug Resistance

Drug resistance and subsequent treatment failure represent a major clinical challenge. Lysosomes have also been described to contribute to resistance to antineoplastic drugs. There are several plausible explanations of this phenomenon, one of which involves the lysosomal sequestration of chemotherapeutic agents [57], which prevents binding to target molecules and thus impairs their cytotoxic activity. The above mentioned mechanism affects mainly lipophilic weak-base drugs, which become trapped inside the lysosomal lumen after protonation inside acidic environment. Moreover, it has been also postulated that some of the lysosome-accumulating agents, such as doxorubicin and TKIs, upregulate the biogenesis of lysosomal compartment, further enhancing lysosomal drug sequestration and therapy resistance [50,105,106,107,108]. Another report suggests that lysosomal accumulation of drugs stimulate exocytosis of the lysosomal content [109], thereby contributing to drug transportation outside the cell through a mechanism different than multidrug resistance (MDR) efflux transporters of the ATP-binding cassette (ABC) superfamily [110]. Therefore, the use of lysosomotropic agents seems an attractive solution to overcome the problem of their lysosomal-dependent drug resistance [50]. Indeed, CQ significantly increased the concentration of imatinib outside the lysosome in a murine bone marrow-derived cell line, suggesting that lysosome targeting can improve the efficacy of TKIs [50].

## 5. Concluding Remarks and Future Directions

Despite a significant progress in cancer treatment in the recent years, a high proportion of patients still develops drug resistance and relapses. Therefore, there is a constant need for new therapeutic approaches. Given the altered lysosomal biology in cancer cells, lysosome-targeting agents represent a promising antitumor strategy. Indeed, therapies involving various lysosome-targeting drugs alone or in combination with other chemotherapeutics show remarkable antineoplastic efficacy in various in vitro and in vivo studies and the list of agents interfering with lysosomal compartment constantly expands. Unfortunately, the utility of the lysosome-targeting agents, as anticancer drugs, can be limited by their low cancer selectivity, which results in substantial toxicity. Therefore, it is necessary to search for novel agents, which would enable specific targeting of lysosomes in cancer cells. Moreover, it is worth to highlight that lysosomes are engaged in shaping a response to various anticancer regimens even in circumstances, where the primary mechanism of cell death is different from LCD. Therefore, combining these therapeutic modalities with typical lysosomotropic agents could be potentially beneficial and would be interesting to be tested in preclinical studies. Moreover, targeting lysosomes represents a promising and feasible approach to overcome drug resistance. Further studies are needed to investigate the clinical utility and efficacy of lysosome-targeting agents in cancer patients.

Lysosome membrane integrity is protected by HSP70 as well as lysosomal-associated membrane protein 1/2 (LAMP1/2), LIMP2, and CD63. Accordingly, degradation of LAMP1 and HSP70 leads to lysosome membrane permeabilization (LMP). LMP may be also induced by other stimuli, including ROS (H_2_O_2_), proteases such as caspases and calpains, cytoskeleton disruption and changes in sphingolipids composition in lysososmal membrane, e.g., increase in sphingomyelin and sphingosine. Inhibition of V-type H^+^ ATPase and therefore impaired acidification of the lysosome is also contributing to its destabilization. As a result of these events, the breakdown of lysosomal membrane provokes cathepsins release and subsequently lead to cell death. Detailed characteristic of LMP-promoting mechanisms are described in paragraph 2. The LMP-inducing mechanisms are displayed in rectangles.

## Figures and Tables

**Table 1:** List of typical inducers of lysosomal cell death.

Drug	Combination/Monotherapy	Study Model	Mechanism	Reference
Chloroquine and its derivates				
Chloroquine	In combination with tyrosine kinase inhibitors (TKIs)	Chronic myelogenous leukemia (CML)	Prevention of lysosomal sequestration through lysosomal membrane permeabilization (LMP)	[50]
Chloroquine	Monotherapy	Burkitt’s lymphoma	LMP, autophagy inhibition, p53-dependent cell death	[44]
Mefloquine	Monotherapy	Acute myeloid leukemia (AML)	LMP, reactive oxygen species (ROS) generation	[51]
Hydroxy-chloroquine	Monotherapy	Various cancer cell lines	LMP, followed by MMP and caspase activation	[52]
V-type H^+^ ATPase inhibitors				
Bafiliomycin	Monotherapy	Gastric cell line	Cathepsin release, LMP, caspase-3 dependent cell death	[53]
Omeprazole	Monotherapy	Human lymphoma and leukemia cell lines	Lysosomal alkalization leading to LMP, ROS generation and caspase-independent apoptosis	[37,54]
Heat shock protein 70 (HSP70) inhibitors				
Pifithrin-μ	Monotherapy	Primary effusion lymphoma (PEL)	LMP, mitochondrial depolarization, dendritic cell activation	[55]
Pifithrin-μ	Monotherapy and in combination with cytarabine, 17-(allylamino)-17-desmethoxygeldanamycin, suberoylanilide hydroxamic acid, and sorafenib	AML B-cell acute lymphoblastic leukemia (B-ALL) T-cell acute lymphoblastic leukemia (T-ALL) CML	Apoptosis, cell cycle arrest, caspase-3 activation	[56]
Drugs interfering with sphingolipid metabolism				
Siramesine Nortriptyline Desipramine	Monotherapy	Chronic lymphocytic leukemia (CLL)	LMP, rtrad transcription factor EB (TFEB) nuclear translocation, mitochondrial depolarization, ROS generation, lipid peroxidation, altered sphingosine metabolism	[17]
Siramesine	Monotherapy	Breast and cervical cancer cell lines, murine fibroblasts	LMP, increased ROS generation and nonapoptotic cell death	[15]
	In combination with lapatinib	Human breast cancer cell lines	Increased ROS generation and ferroptosis	[57]
Opaganib (ABC294640)	Monotherapy	Kidney, breast and prostate cancer cell lines	Cell death associated with increased lysosomal size and acidification, potentiated in combination with autophagy inhibitors	[58]

**Table 2:** List of atypical inducers of lysosomal cell death.

Drug	Combination/Monotherapy	Study Model	Mechanism	Reference
Classical chemotherapeutics				
Vincristine	In combination with siramesine	Human breast and cervical cancer cell lines	Increased LMP and synergistic cell death	[25]
Docetaxel	Monotherapy	Prostate cancer cell lines	Cell death partially dependent on LMP	[59]
Fludarabine	Monotherapy and in combination with valproic acid	CLL	LMP and cathepsin B upregulation	[60]
Cisplatin	Monotherapy or in combination with trichostatin A	Head and neck squamous cell carcinoma (SCC)	LMP associated with cathepsin B-mediated LAMP-2 degradation, which could be further potentiated by Trichostatin A treatment	[61]
Bortezomib	Monotherapy	Human pancreatic cancer cells	LMP followed by cathepsin B-mediated activation of caspase 2 and subsequent mitochondrial depolarization	[62]
Tyrosine-kinase inhibitors and BH3-mimetics				
Imatinib	Monotherapy	CML cell lines and CD34+ cells from CML patients	LMP and cathepsin B release into the cytoplasm	[63]
Sorafenib	Monotherapy	Human bladder cancer cell lines	LMP followed by MMP and apoptosis	[64]
Obatoclax	Monotherapy or in combination with chloroquine or bafiliomycin	Thyroid cancer cells	LMP and autophagy blockade	[65]
Monoclonal antibodies				
Anti-CD20 mAbs-tositumomab and obinutuzumab	Monotherapy	Lymphoma and leukemia cell lines	LMP initiated by actin cytoskeleton reorganization upon mAb-mediated homotypic aggregation of cells	[34,66,67]
Anti-CD38 antibodies—daratumumab and isatuximab	Monotherapy	Myeloma cell lines		[68,69]
Photodynamic therapy				
Photosensitizer-NPe6	Monotherapy	Murine hepatoma cell line	LMP and subsequent apoptosis through Bid truncation	[70]

## Abbreviations

ABC	ATP-binding cassette
AML	Acute myeloid leukemia
ASM	Acid sphingomyelinase
B-ALL	B-cell acute lymphoblastic leukemia
BCL-2	B-cell lymphoma 2
BH3	B cell lymphoma-2 (BCL-2) homology domain 3
CAD	Cationic amphiphilic drugs
CLEAR	Coordinated Lysosomal Expression and Regulation
CLL	Chronic lymphocytic leukemia
CML	Chronic myelogenous leukemia
CQ	Chloroquine
HCQ	Hydroxychloroquine
HDAC	Histon deacytelases
HSP70	Heat shock protein 70
LAMP1	Lysosome associated membrane protein 2
LAMP2	Lysosome associated membrane protein 2
LCD	Lysosomal cell death
LIMP2	Lysosome integral membrane protein 2
LMP	Lysosomal membrane permeabilization
mAbs	Monoclonal antibodies
MDR	Multidrug resistance
mTOR	Mammalian target of rapamycin
mTORC1	Mechanistic target of rapamycin complex 1
NADPH	Nicotinamide adenine dinucleotide phosphate
NPe6	Mono-L-aspartyl chlorine e6
PARP	Poly (ADP-ribose) polymerase
PDT	Photodynamic therapy
PEL	Primary effusion lymphoma
ROS	Reactive oxygen species
SCC	Squamous cell carcinoma
T-ALL	T-cell acute lymphoblastic leukemia
TFEB	Transcription factor EB
TKIs	Tyrosine-kinase inhibitors
